# Evaluation of three recombinant proteins for the development of ELISA and immunochromatographic tests for visceral leishmaniasis serodiagnosis

**DOI:** 10.1590/0074-02760180405

**Published:** 2019-02-04

**Authors:** Anna Raquel Ribeiro dos Santos, Ângela Vieira Serufo, Maria Marta Figueiredo, Lara Carvalho Godoi, Jéssica Gardone Vitório, Andreza Pain Marcelino, Daniel Moreira de Avelar, Fernandes Tenório Gomes Rodrigues, George Luiz Lins Machado-Coelho, Fernanda Alvarenga Cardoso Medeiros, Selma Maria Bezerra Jerônimo, Edward José de Oliveira, Frederico Crepaldi Nascimento, Santuza Maria Ribeiro Teixeira, Ricardo Tostes Gazzinelli, Ronaldo Alves Pinto Nagem, Ana Paula Fernandes

**Affiliations:** 1 Universidade Federal de Minas Gerais, Faculdade de Farmácia, Belo Horizonte, MG, Brasil; 2 Fundação Oswaldo Cruz-Fiocruz, Centro de Pesquisas René Rachou, Belo Horizonte, MG, Brasil; 3 Centro de Tecnologia em Vacinas da Universidade Federal de Minas Gerais, Belo Horizonte, MG, Brasil; 4 Universidade Federal de Minas Gerais, Departamento de Bioquímica e Imunologia, Belo Horizonte, MG, Brasil; 5 Universidade Federal de Ouro Preto, Departamento de Medicina de Família, Saúde Mental e Coletiva, Ouro Preto, MG, Brasil; 6 Fundação Ezequiel Dias, Instituto Octávio Magalhães, Belo Horizonte, MG, Brasil; 7 Universidade Federal do Rio Grande do Norte, Departamento de Bioquímica, Natal, RN, Brasil

**Keywords:** visceral leishmaniasis, recombinant proteins, diagnosis, ELISA, immunochromatographic test

## Abstract

**BACKGROUND:**

Visceral Leishmaniasis (VL) is an infectious disease that is a significant cause of death among infants aged under 1 year and the elderly in Brazil. Serodiagnosis is a mainstay of VL elimination programs; however, it has significant limitations due to low accuracy.

**OBJECTIVE:**

This study aimed to evaluate three recombinant *Leishmania infantum* proteins (rFc, rC9, and rA2) selected from previous proteomics and genomics analyses to develop enzyme-linked immunosorbent assay (ELISA) and immunochromatographic tests (ICT) for the serodiagnosis of human VL (HVL) and canine VL (CVL).

**METHODS:**

A total of 186 human (70 *L. infantum*-infected symptomatic, 20 other disease-infected, and 96 healthy) and 185 canine (82 *L. infantum*-infected symptomatic, 27 *L. infantum*-infected asymptomatic, and 76 healthy) sera samples were used for antibody detection.

**FINDINGS:**

Of the three proteins, rA2 (91.5% sensitivity and 87% specificity) and rC9 (95.7% sensitivity and 87.5% specificity) displayed the best performance in ELISA-HVL and ELISA-CVL, respectively. ICT-rA2 also displayed the best performance for HVL diagnosis (92.3% sensitivity and 88.0% specificity) and had high concordance with immunofluorescence antibody tests (IFAT), ELISA-rK39, IT-LEISH^®^, and ELISA_EXT_. ICT-rFc, ICT-rC9, and ICT-rA2 had sensitivities of 88.6%, 86.5%, and 87.0%, respectively, with specificity values of 84.0%, 92.0%, and 100%, respectively for CVL diagnosis.

**MAIN CONCLUSIONS:**

The three antigens selected by us are promising candidates for VL diagnosis regardless of the test format, although the antigen combinations and test parameters may warrant further optimisation.

Visceral Leishmaniasis (VL), also known as kala-azar, continues to be a deadly infectious disease and a global public health problem. *Leishmania donovani* is associated with the anthroponotic transmission of VL in Asian and African countries, whereas *Leishmania infantum* is the etiologic agent of zoonotic VL in countries of the Mediterranean Basin and Latin America. These parasites are transmitted to humans and other mammals by the bite of sandflies from the genera *Phlebotomus* (Old World) and *Lutzomyia* (New World).^1^ Dogs are the main urban and peridomestic source of *L. infantum* parasites due to their high rates of infection, high parasite densities, and ability to transmit infection even when asymptomatic. Human VL (HVL) and canine VL (CVL) may be lethal if not promptly diagnosed and treated. Symptoms, which can be confounded with manifestations of several other diseases, are fever, weight loss, splenomegaly, hepatomegaly, and anaemia. In symptomatic dogs, several concomitant signs of infection include cachexia, alopecia, onychogryphosis, skin ulcers, and dermatitis.^2^ ^,^ ^3^

Worldwide implementation and management of strategies for the prevention and control of VL remain insufficient, resulting in sustained high mortality rates and geographical expansion of the disease to previously unreported areas. In Brazil, significant changes in VL control and surveillance programs have been implemented, including the screening of infected dogs with the immunochromatographic test (ICT) DPP^®^-CVL (Dual-Path Platform - Bio-Manguinhos/Fiocruz, Rio de Janeiro, Brazil) followed by an enzyme-linked immunosorbent assay (ELISA) using crude parasite extracts as a confirmatory test.^4^

Detection of parasite-specific antibodies through ICT is a promising alternative because the technique is simple and rapid; in contrast, parasitological and molecular tests require invasive tissue sampling, trained personal, and laboratory equipment. However, considerable variation in sensitivity values for the detection of both HVL and CVL has been reported for ICT, indicating that there is still much room for improvement.

The recombinant kinesin repetitive (rK) antigens are the most widely used antigens in commercial ICTs. The rK39-ICT antigen displays good sensitivity in symptomatic cases of CVL, but lacks sensitivity (ranging from 52.9% to 77%) in the diagnosis of asymptomatic dogs.^5^ ^,^ ^6^ Similarly, the sensitivity reported for DPP^®^-CVL with the rK28 antigen was between 47% and 92% among asymptomatic CVL cases.^7^ ^,^ ^8^

In this context, genomic and proteomic approaches combined with bioinformatics should allow for the discovery of new *Leishmania* antigenic proteins, the improvement of available diagnostic tests, and the development of new tests. Among the candidate antigens, the *L. infantum* and *L. donovani* amastigote-specific A2 antigen is similar to the kinesins and contains a repetitive amino acid sequence. The A2 antigen has also been recognised as a promising antigen for the serodiagnosis of VL, displaying excellent specificity (98%) and increased sensitivity in the detection of VL in asymptomatic dogs (88%) than rK39 or rK26 (both 66%).^9^ ^,^ ^10^ ^,^ ^11^

The Sec 14 cytosolic factor (Fc), encoded by the *L. infantum* gene LinJ36_V3.0640, promotes the transport of proteins through the Golgi complex. The gene encoding Fc is present in the *L. infantum* genome but is a pseudogene in *Leishmania braziliensis* and is absent in the *Leishmania major* genome.^12^ The C9 antigen has been identified through immunoproteomics of *L. infantum* promastigote extracts as a hypothetical protein (GI 146076809).^13^ When tested in ELISA, the recombinant C9 protein (rC9) displayed an overall sensitivity of 68% and specificity of 78% with human sera samples as well as 70.6% sensitivity and 82% specificity for the detection of VL in dog sera samples. However, rC9 detected 92.8%^14^ and 94.8%^15^ of the samples from asymptomatic dogs. Therefore, the C9 antigen requires further validation as a target for VL diagnosis.

Given their characteristics and previously reported potential, this study evaluated A2, Fc and C9 as diagnostic antigens to develop high performance ELISA and ICT for canine and human VL.

## MATERIALS AND METHODS

*Ethics statement* - The tests involving canine and human samples were conducted in agreement with the Ethical Principles in Animal and Human Research and were approved by the Ethics Commission on Animal Use/ UFMG (Protocol: 298/2016) and Research Ethics Committee/UFMG (CAAE: 67820516.8.1001.5149).

*Sample size estimation* - To calculate the numbers of canine and human samples, expected sensitivities (95% and 96%, respectively) and specificities (95% and 96%, respectively) were considered. Based on these parameters, the sample size was calculated using the following equation: n ≥ (1.96)^2^. p (1 - p) / x^2^, where n = positive or negative numbers, p = sensitivity (or specificity) index, and x = 0.05, resulting in a minimum number of positive or negative samples.

*Canine sera samples* - Sera samples of dogs (n = 185) from different regions of the state of Minas Gerais, Brazil (metropolitan region of Belo Horizonte and municipalities of Porteirinha and Ouro Preto) were used in this study to determine the specificities and sensitivities of the ELISA and ICT assays using the three recombinant proteins. These samples were collected during clinical trials (15%), at veterinary hospitals (22%), or from the Centres of Zoonosis Control (63%) (Table I). Based on previous results of diagnostic tests, of the total samples, 59% (n = 109) were positive for CVL, while 41% (n = 76) of the samples were negative. For diagnosis, the *L. infantum*-infected dogs were examined by veterinarians and were classified as symptomatic if the animals presented typical signs of CVL (alopecia, dermatitis, conjunctivitis, lymphadenopathy, onychogryphosis, etc.) or asymptomatic (0-2 typical signs or absent signs). Among the positive samples, 109 corresponded to *L. infantum*-infected symptomatic (n = 82) or asymptomatic (n = 27) animals, and 76 were from healthy dogs. Clinical conditions among symptomatic animals were heterogeneous. All animals were tested for parasite detection [direct optical microscopy and/or parasite culture and/or polymerase chain reaction (PCR)] and subjected to serological tests. Serological tests, including EIE-CVL (Bio-Manguinhos/Fiocruz), the immunofluorescence antibody test (IFAT), ICT- Alere^®^ (Bionote Inc., Korea), or DPP^®^-CVL were also used to characterise the canine sera samples. The diagnostic criteria (reference standard) were based on the positivity of at least one parasitological test (direct parasitological test or parasite culture) or PCR, and one serological positive result (Table II).

**TABLE I:** Sources of dog samples

Source of sample	Diagnosis criteria		Total n (%)
	Positive n (%)^*^	Negative n (%)^**^	
Center for Zoonosis Control^*a*^	62 (57)	55 (73)	117 (63)
Veterinary hospital^*b*^	27 (25)	13 (17)	40 (22)
Clinical trials^*c*^	20 (18)	8 (10)	28 (15)
Total	109 (100)	76 (100)	185 (100)

a: metropolitan region of Belo Horizonte and municipalities of Ouro Preto; b: metropolitan region of Belo Horizonte; c: municipalities of Porteirinha and Ouro Preto; *: positive by direct parasitology, culture or polymerase chain reaction (PCR); **: negative by serology.

*Human sera samples* - VL patients consisted of women (35.8%) and men (64.2%), with an average age of 18.2 years. All *L. infantum*-infected patients presented with clinical symptoms of VL (n = 70). Healthy donors included in the study did not display any leishmaniasis symptoms on the date the blood samples were collected (n = 96). Also, they did not display signs suggestive of any other infectious disease and were not on medication for any chronic disease. These samples were obtained from the Central Public Health Laboratory (CPHL) at the municipalities of Palmas (Tocantins state, Brazil), Belo Horizonte (Minas Gerais state, Brazil) and Natal (Rio Grande do Norte state, Brazil). ELISAs were performed for all HVL samples using total extracts of *L. infantum* (ELISA_EXT_) at the René Rachou Institute (Fiocruz - Minas Gerais) and the recombinant protein K39 (ELISA-rK39) at the Federal University of Rio Grande do Norte. The samples were also tested using the commercial immunochromatographic tests IT-LEISH^®^ (Bio - Rad Laboratories, Inc.) at the Federal University of Minas Gerais. Positive results of all serological tests were used as reference standards. All samples were retested for serological diagnosis. These assays were performed identically across all negative and positive samples at the Center of Vaccine Technology (UFMG), except for the ELISA_EXT_, which was performed at the René Rachou Institute (Fiocruz - Minas Gerais). Infection by *L. infantum* in VL patients was confirmed either by parasitological detection (culture or direct microscopic examination) or PCR in a set of 10 samples.

**TABLE II:** Summary of results of canine samples using different laboratory diagnostic tests

	Positive samples (n = 109)^*a*^	Negative sample (n = 76)^*b*^
Laboratory test	n^*c*^ (%)	n^*d*^ (%)
Direct parasitology	51 (47)	73 (96)
Culture	47 (43)	38 (50)
PCR	35 (32)	32 (42)
Total	109 (100)	76 (100)

a: all animals (n = 109) were positive in all serological tests; b: all healthy animals (n = 76) were negative in all serological tests; c: number of positive results in respective diagnostic tests; d: number of negative results in respective diagnostic tests.

For evaluation of cross-reactivity with other diseases, sera from patients previously diagnosed with Chagas disease (n = 5), malaria (n = 5), toxoplasmosis (n = 5) or American Tegumentary Leishmaniasis (ATL) (n = 5) were also included*.* Sera from VL patients (n = 19) and healthy donors (n = 5) were included in this assay as positive and negative controls, respectively.

Additionally, a subset of samples obtained from VL patients (n = 50) and healthy subjects negative for VL (n = 37) were evaluated with a commercial kit available for Chagas disease diagnosis (ELISA Chagas III-Grupo Bios S.A - Chile).

*Expression and purification of recombinant proteins Fc, C9 and A2* - The rFc protein was obtained after the PCR amplification and cloning of its coding region from the genome of *L*. *infantum* BH46 (MHOM/BR/1975/M2682). For this process, genomic DNA was extracted from promastigotes growing in Schneider’s medium (Sigma-Aldrich) and PCR amplification was performed using specific forward (5’-CTTCATATGGCGGCAACTCATCTTACC-3’) and reverse (5’-CATGGATCCTCACTTCGGCAAACCGTT-3’) primers. The amplified PCR product was digested using *BamHI/Ndel* restriction enzymes and cloned into the pET15B vector (Novagen) before being transformed into *Escherichia coli* (C41 strain) host cells for protein expression. To promote protein expression, bacterial cells transformed with the plasmids were grown in LB (Luria-Bertani) medium with 100 µg/mL ampicillin and were induced with 1 mM IPTG (isopropyl-β-D-thiogalactopyranoside) (Sigma-Aldrich) at 37ºC for 3 h. The recombinant protein was purified through affinity chromatography using a His-Trap column (GE Healthcare Life Sciences), according to the manufacturer’s protocol.

The rC9 protein was originally identified during an analysis of *L. infantum* proteins by two-dimensional gel electrophoresis and immunoproteomics.^13^ Based on its gene sequence, primers were designed for PCR amplification from genomic DNA extracted from *L. infantum* (MHOM/BR/1972/BH46). The PCR-amplified DNA was cloned into a pET - 28a - TEV vector and subsequently transformed into *E. coli* (BL 21 strain) host cells. Transformed cells were grown in 2xYT medium (1.6% tryptone, 1% yeast extract, 0.5% NaCl) with 0.05 mg/mL kanamycin and induced with 0.5 mM IPTG (Sigma-Aldrich) for expression at 37ºC for 4 h. Recombinant protein was purified by affinity chromatography using a His-Trap column (GE Healthcare Life Sciences).^14^

For rA2 production, a codon-optimised gene containing the sequence spanning the coding region of the A2 protein was synthesised.^16^ This sequence contained 10 repeats present in the A2 gene and codons for the C-terminal six-residue histidine tag. The endogenous *L. infantum* A2 genes (XM_001465551) encode proteins containing 40-90 repeated units.^10^ The synthetic gene was cloned into the pET9a vector (Novagen) to generate the pET9a24a-A2His plasmid. Next, *E. coli* (C41 strain) cells were transformed with the recombinant plasmid. Transformed cells were grown in glucose and yeast extract medium containing kanamycin (100 µg/mL) and chloramphenicol (36 µg/mL) using a BIOSTAT B Plus fermenter (Sartorius), and expression was induced by 1 mM IPTG (Sigma-Aldrich). Subsequently, cells were centrifuged and lysed by sonication. The A2 protein was purified under denaturing conditions using a His-Trap column (GE Healthcare Life Sciences) followed by a second purification step using a HiTrap^TM^ Desalting column (GE Healthcare Life Sciences) according to the manufacturer’s protocol.

Purified rFc, rC9 and rA2 proteins were analysed by dodecyl sulphate-polyacrylamide gel electrophoresis (SDS-PAGE) and quantified using the 2D Quant Kit (GE Healthcare Life Sciences).

*Specific antibody levels in canine and human samples* - In canine samples, serum levels of IgG specific for each of the three antigens were detected using ELISA plates (Costar^®^) coated with rA2 (0.062 µg/well), rFc (0.125 µg/well) or rC9 (0.062 µg/well) diluted in carbonate-bicarbonate buffer (pH 9.6). In human samples, rA2 (0.2 µg/well), rFc (0.1 µg/well) or rC9 (1 µg/well) were used. Plates were incubated with the antigens at 4ºC for 18 h. After blocking with 1% bovine serum albumin (200 µL/well) at 37ºC for 1 h, serum samples (100 µL/well) were added at a final dilution of 1:100 and incubated at 37ºC for 1 h. Antibody-antigen binding was detected by the addition of peroxidase-conjugated goat anti-dog IgG (1:25.000) or peroxidase-conjugated anti-human IgG (1:100.000). The presence of bound IgG was detected using O-phenylenediamine dihydrochloride (Sigma-Aldrich) with H_2_O_2_ diluted in 0.05 M citrate-phosphate buffer (pH 5.0) and stopped by the addition of 2 N H_2_SO_4_. Optical density (OD_492_) values were obtained using a Multiskan GO microplate spectrophotometer (Thermo Fisher Scientific).

The ELISA Chagas III kit (Grupo Bios S.A. - Chile) was used according to manufacturer’s specifications.

*Immunochromatographic test* (ICT) - The ICT uses the principle of lateral flow and consists of a piece of nitrocellulose membrane (for immobilising the test and control lines), glass fibre (containing the conjugate - colloidal gold), cellulose fibre (to absorb the sample), and a plastic cassette. To compose the test lines, nitrocellulose membranes were impregnated with rFc, rC9 or rA2 using a Jet Spray (EASE-Medtrend Biotech, China). A polyclonal antibody produced in rabbits immunised with each recombinant protein was also sprayed onto the membrane to obtain the control line. For conjugation, protein was mixed with colloidal gold (Sigma-Aldrich) and incubated at room temperature for 20 min. The conjugate solution was stabilised with 1% BSA (Sigma-Aldrich) and then centrifuged. The supernatant was discarded, and the pellet was resuspended in storage buffer, adsorbed to the glass fibre, and dried in a low humidity room.

To perform the ICT, 5 µL of each serum sample was mixed with the running buffer and applied over the ICT strip. If a sample contained anti-*L. infantum* antibodies, the antibodies first reacted with the antigen-gold colloid conjugates in the conjugation pad. As the antibody-antigen gold colloid complex flowed past the capture site, the antibodies reacted with the antigens at the site of the test line, leading to the formation of a visible red line within 10-15 min. In the absence of specific antibodies (negative samples), no reactivity was observed at this site.

*Statistical analysis* - Statistical analysis was performed using the GraphPad Prism 5.01 software. Receiver operating characteristic (ROC) curves were constructed to determine the cut-off values and to estimate the sensitivity (Se), specificity (Sp), and confidence intervals (95% CI) of each assay. Cut-off values were used to discriminate between the numbers of true positive (TP) and true negative (TN) samples. Accuracy was calculated using the sum of TP and TN samples divided by the total number of tested samples.

The concordance between ELISA and ICT for each antigen and ICT-HVL with each antigen versus IFAT, IT-LEISH^®^, ELISA_EXT_ and ELISA-rK39 was calculated using the Kappa (k) index, according to Cohen. McNemar’s test was used to estimate statistical differences between pairs of tests. Differences were considered statistically significant when the *p* value < 0.05.

## RESULTS

**Fig. 1:**
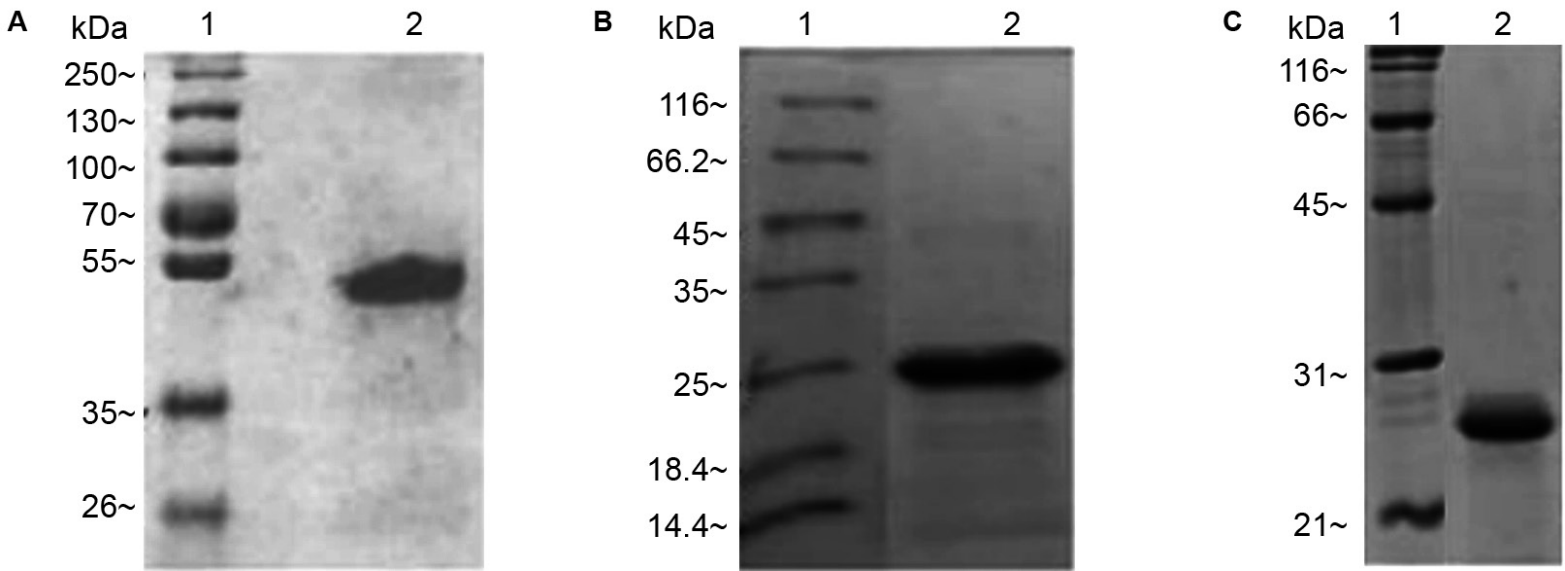
dodecyl sulphate-polyacrylamide gel electrophoresis (SDS-PAGE) analysis of recombinant proteins Fc, C9, and A2 for purification. Electrophoretic analysis (A) molecular weight marker (line 1) and purified rFc (line 2) (~55 kDa), (B) molecular weight marker (line 1) and purified rC9 (line 2) (~24.4 kDa), and (C) molecular weight marker (line 1) and purified rA2 (line 2) (~25 kDa).

The reactivity of specific antibodies present in canine samples was evaluated by ELISA using the purified rFc (Fig. 1A), rC9 (Fig. 1B) and rA2 (Fig. 1C) recombinant antigens. ELISA was performed on 109 samples from symptomatic (n = 82) and asymptomatic (n = 27) CVL dogs and 76 samples from healthy dogs. ROC curves were generated for rFc, rC9, and rA2 to determine the test sensitivity and specificity. An excellent performance was observed for each antigen, corresponding to Se values of 93.6%, 95.7%, and 93.6% and Sp values of 82.3%, 87.5%, and 81.2% for rFc, rC9, and rA2, respectively, as shown in Table III. Regarding the detection of symptomatic CVL cases, the rFc, rC9, and rA2 proteins showed Se values of 92.6%, 96.3%, and 96.3%, whereas the Se values for asymptomatic cases were 95%, 90%, and 90%, respectively (Table III). The best accuracy was obtained with rC9 using ELISA-CVL (93.6%).

Similarly, we evaluated the performance of rFc, rC9, and rA2 for ELISA to detect antibodies in sera from HVL patients. ROC curves were generated, and the optimal cut-off point was selected to determine the Se and Sp for each protein. Antibodies against rFc, rC9, and rA2 were found in 84.2%, 87.2%, and 91.5% of the positive sera samples, respectively, validating the rA2 protein as an excellent candidate for the diagnosis of HVL; rA2 provided the best accuracy (87.5%) among all proteins (Table III). Among the 96 negative samples tested, 87% were negative in the ELISA-rA2, whereas 72% and 67% of the samples tested in the ELISA-rFc and ELISA-rC9, respectively, displayed negative results when tested with these antigens (Table III).

**Fig. 2:**
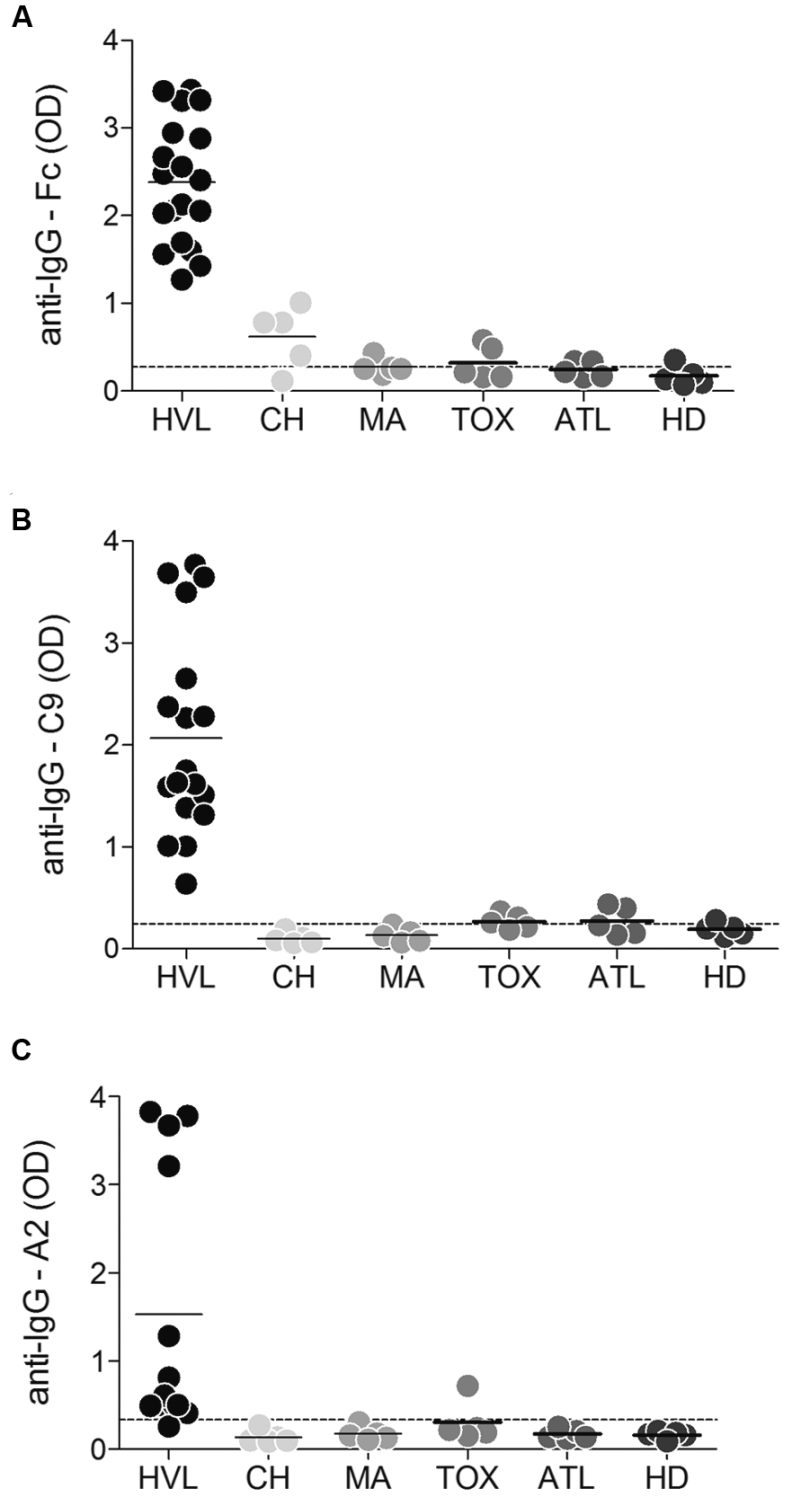
antibody levels in sera of patients with human visceral leishmaniasis (HVL), patients with other diseases, and healthy subjects. (A) the Fc protein: 0.1 µg/well; (B) the C9 protein: 1 µg/well; and (C) the A2 protein: 0.2 µg/well were tested by ELISA for anti-Fc, anti-C9, and anti-A2 antibodies. The samples were separated in groups as follows: sera from patients with VL (n = 19), Chagas disease (CH) (n = 5), Malaria (MA) (n = 5), Toxoplasmosis (TOX) (n = 5), American Tegumentary Leishmaniasis (ATL) (n = 5), and healthy subjects (HD) (n = 5). The cut-off values (dotted lines) were calculated using ROC curves positive samples (HVL) versus negative samples (healthy donors).

**Fig. 3:**
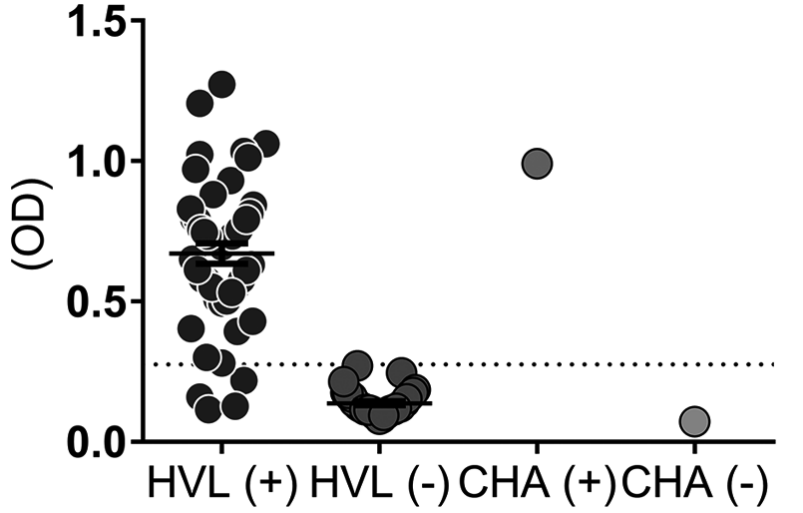
commercial ELISA (Chagas III). The commercial Chagas III Kit was used to test 50 patients with VL (HVL +), 37 healthy subjects, subjects negative for VL (HVL-), control positive kit (CHA+) and control negative kit (CHA-). Dotted lines represent the cut-off. The cut-off values (dotted lines) were calculated using ROC curves positive samples (HVL+) versus negative samples (HVL-).

*L. infantum*-infected samples were also compared with sera of healthy donors and patients with other infections (Fig. 2). Among the samples of patients with other diseases, four out of five samples from Chagas disease patients, one sample from a malaria patient, and two samples each from the toxoplasmosis and ATL groups of patients were positive for rFc; two and three samples from the ATL and toxoplasmosis groups, respectively, were positive for rC9; and only one sample from a toxoplasmosis patient was positive for rA2. Interestingly, a subset of samples obtained from VL patients (n = 50) displayed 92% cross-reactivity in ELISA using the Chagas disease commercial kit. This kit, however, displayed 100% Sp with sera from negative, healthy subjects (n = 37) (Fig. 3).

**Fig. 4:**
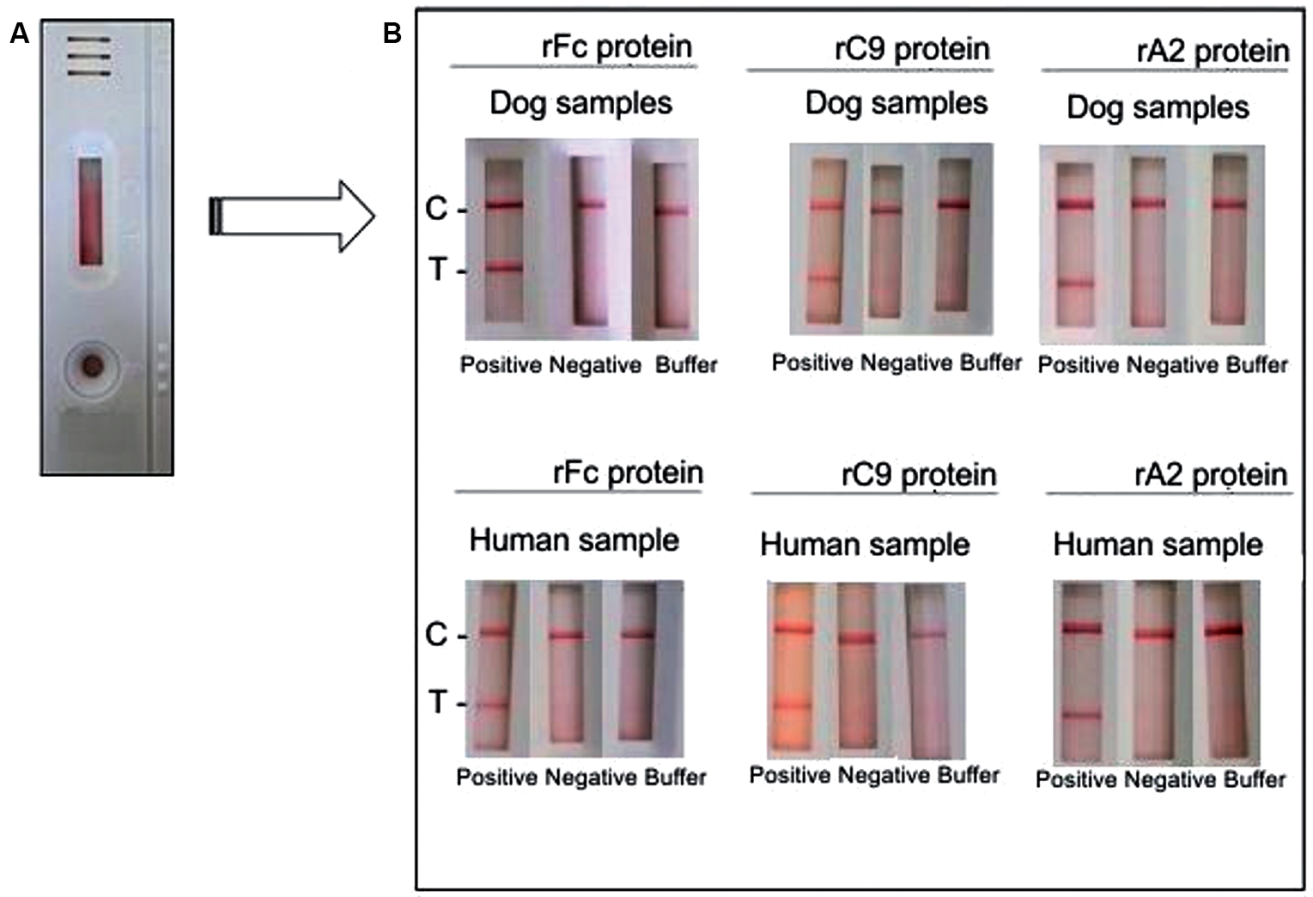
representation of the positive and negative results of ICT-rFc, ICT-rC9 and ICT-A2. (A) Representative test (B) with dog serum and human serum: the presence of both the test line (T) and control line (C) represent a positive result upon application of a VL-positive dog or human serum. The presence of the control line only represents a negative result with sample buffer or with VL-negative dog or human serum.

We next assessed the presence of total specific anti-rFC, anti-rC9 and anti-rA2 IgG antibodies in the sera of CVL and HVL cases in ICT. Results were only considered if the reactivity of the control line was observed in ICT. The colour intensity of the test line was dependent on the concentration of the antibody present in each sample, indicating reactivity with each recombinant protein. If both lines were detected, the sample was considered positive. The presence of a signal only in the control line indicated the absence of specific antibodies (Fig. 4).

In ICT, each protein was tested against sera from 109 dogs with VL and 76 healthy donors to determine Se and Sp. The ICT-rFc, ICT-rC9, and ICT-rA2 had Se values of 88.6%, 86.5%, and 87% with Sp values of 84%, 92%, and 100%, respectively (Table IV). The ICT versus ELISA tests using canine samples displayed good concordance for rFc (k = 0.93), rC9 (k = 0.8), and rA2 (k = 0.76). By applying McNemar’s test, there was no statistical difference between ICT-rFc and ELISA-rFc; however, differences were observed between the ICT-rC9 and ELISA-rC9 (p = 0.015) and the ICT-rA2 and ELISA-rA2 (p < 0.001) (Table V).

We also assessed the performance of ICTs prepared with each recombinant protein to detect the presence of total IgG in human sera, which were previously characterised by IFAT, IT-LEISH^®^, ELISA-rK39, and ELISA_EXT_. For this, 70 samples from HVL cases and 96 negative sera samples were used. As shown in Table IV, Se values of 88.6%, 78.6% and 92.3% and Sp of 64.1%, 84% and 88%, were observed for ICT-rFc, ICT-rC9 and ICT-rA2, respectively.

**TABLE III:** Performance of enzyme-linked immunosorbent assay (ELISA) for diagnosis of canine visceral leishmaniasis (CVL) and human VL (HVL) using the rFc, rC9 and rA2 antigens

Visceral leishmaniasis^*a*^	Antigen	% Se^*b*^ (95% CI)^*c*^	% Sp^*d*^ (95% CI)	% Accuracy^*e*^
Canine	rFc	93.6 (82.5-98.7)	82.3 (56.6-96.2)	90.6
	rC9	95.7 (85.5-99.5)	87.5 (61.6-98.5)	93.6
	rA2	93.6 (82.5-98.7)	81.2 (54.3-96.0)	90.4
		% Se (95% CI) Sym^*f*^	% Se (95% CI) Asym^*g*^	% Accuracy (Sym/Asym)
	rFc	92.6 (75.7-99.0)	95.0 (75.1-99.8)	88.6/89.2
	rC9	96.3 (81.0-99.9)	90.0 (68.3-98.7)	93.2/89.2
	rA2	96.3 (81.0-99.9)	90.0 (68.3-98.7)	90.9/ 86.5
Human	Antigen	% Se	% Sp	% Accuracy
	rFc	84.2 (60.4-96.6)	72.0 (46.5-90.9)	78.3
	rC9	87.2 (66.8-98.7)	67.0 (43.0-85.4)	75.9
	rA2	91.5 (63.9-99.8)	87.0 (73.7-95.0)	87.5

a: positive samples (canine, n = 109 or human, n = 70) and negative samples (canine, n = 76 or human, n = 96); b: Se - sensitivity; c: 95% CI: 95% probability confidence interval; d: Sp - specificity; e: accuracy = True Positives + True Negatives/total of samples; f: Sym - symptomatic; g: Sym - asymptomatic.

Good concordance was observed between the ICTs with rFc (k = 0.69) and rC9 (k = 0.65) and the corresponding ELISA for detection of HVL, whereas the ICT and ELISA with rA2 had high concordance (k = 0.98). By applying McNemar’s test, a significant difference was observed between the ICT-rC9 and the ELISA-rC9 (p < 0.001). In contrast, significant differences were not detected between the ICT-rFc and the ELISA-rFc or the ICT-rA2 and the ELISA-rA2 (Table V).

Moreover, the ICT-rFc and ICT-rC9 had moderate concordance, and ICT-rA2 had good concordance with IFAT (k = 0.57, 0.60, and 0.74), IT-LEISH^®^ (k = 0.47, 0.54, and 0.67), ELISA_EXT_ (k = 0.53, 0.6, and 0.74), and ELISA-rK39 (k = 0.5, 0.58, and 0.77), respectively, according to the kappa index. Using McNemar’s test, a statistical difference was observed between the ICT-rFc and the IFAT (p < 0.001), IT-LEISH^®^ (p = 0.002), ELISA_EXT_ (p = 0.014), and ELISA-rK39 (p = 0.006) tests, and no difference was observed between ICT-rA2 or ICT-rC9 versus the IFAT, IT-LEISH^®^, ELISA_EXT_, and ELISA-rK39 tests (Table VI).

The Sp of the ICTs were also tested against sera of patients diagnosed with other parasitic infections, namely malaria (n = 5), toxoplasmosis (n = 5), and Chagas disease (n = 5). One malaria sample had a false positive result with ICT-rFc. The ICT-rC9 also displayed false positive results with samples from patients with ATL (n = 3), toxoplasmosis (n = 4), and Chagas disease (n = 4), while no false positive results were observed for ICT-A2.

**Fig. 5:**
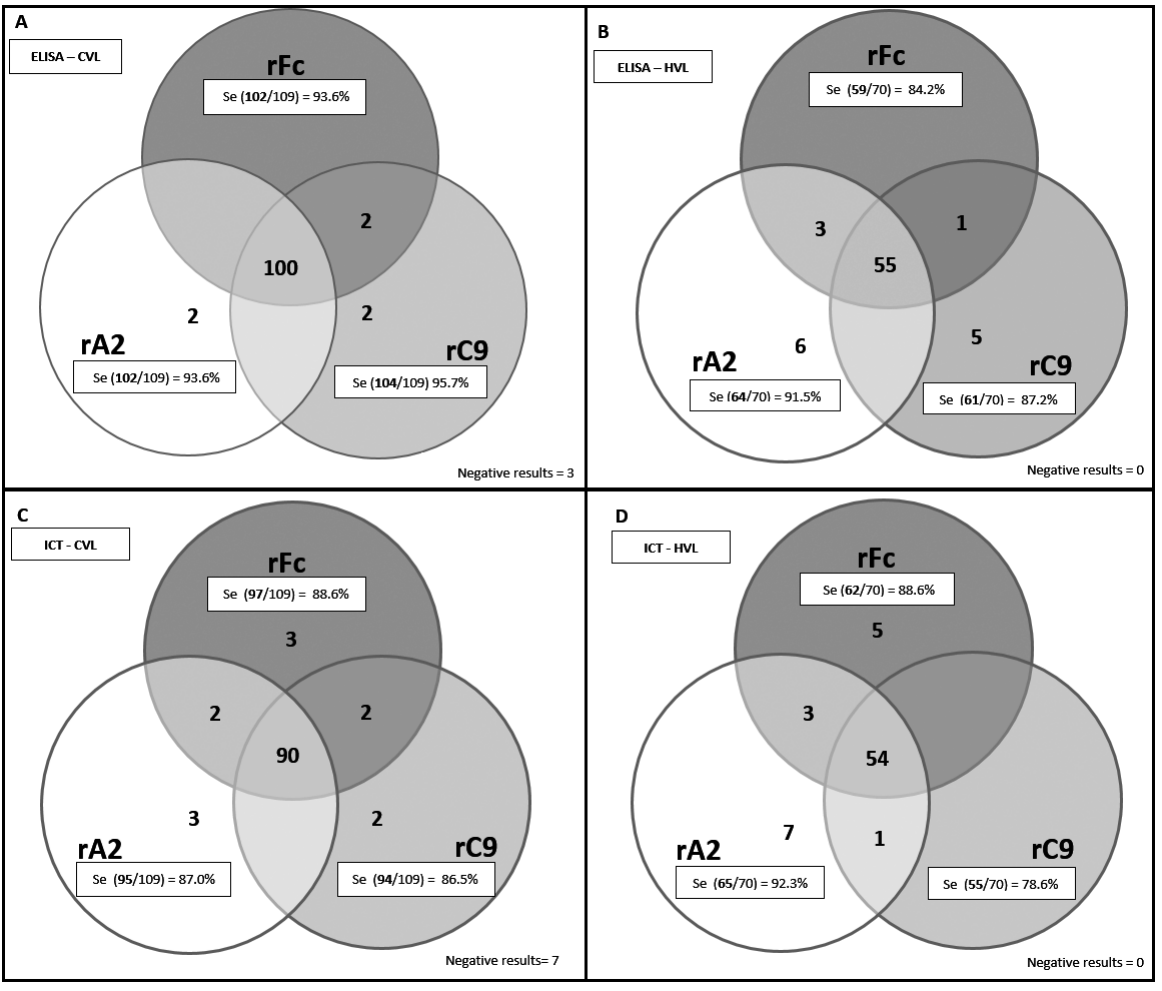
venn diagrams of positive results for the diagnosis of infected dogs (CVL) (n = 109) and human patients (HVL) (n = 70) as detected by ELISA (A and B, respectively) and ICT assays (C and D, respectively) with each antigen (circles). Each circle contains the number of samples with positive results/total samples tested in ELISA or ICT with each antigen and the corresponding sensitivity values. Numbers of samples that were positive in more than one antigen are displayed in circle intersections.

Venn diagrams that compared the two test formats and the three antigens were constructed for positive samples (Fig. 5). This analysis showed that for the ELISA-CVL, a high proportion of samples (100 out of 109) was recognised as positive, regardless of the antigen tested, whereas very few samples were positive for a single antigen (two samples were positive exclusively for rC9 or rA2 or for rFc and rC9) (Fig. 5A). The Venn diagram for ELISA-HVL (n = 70) revealed 55 samples that were positive, regardless of the antigen tested (Fig. 5B). Of the total samples for ELISA-HVL, five samples were positive exclusively for rC9, six samples were positive only for rA2, one sample was positive for rFc and rC9, and three samples were positive for rFc and rA2. For ICT-CVL, 90 samples out of 109 were positive, regardless of the antigen tested (Fig. 5C), and 54 presented a similar pattern for ICT-HVL (Fig. 5D). For ICT-CVL, three, two, and three samples were positive exclusively for rFc, rC9, and rA2, respectively, and two samples were positive either for rA2 and rFc or rFc and rC9. For ICT-HVL, five samples were positive only for rFc, seven samples were positive for rA2, one sample was positive for rC9 and rA2, and three samples were positive for rFc and rA2.

**TABLE IV:** Sensitivity and specificity of ICT-rFc, ICT-rC9 and ICT-rA2 with canine and human sera

	% Se^*a*^ (95% CI^*b*^ ) (n = 109)	%Sp^*c*^ (95% CI) (n = 76)
ICT	Infected dogs	Healthy dogs
rFc	88.6 (75.4-96.2)	84.0 (70.8-92.8)
rC9	86.5 (72.7-94.8)	92.0 (80.8-97.8)
rA2	87.0 (72.6-94.8)	100 (92.9-100)
	% Se (n = 70)	%Sp (n = 96)
ICT	Infected humans	Healthy humans
rFc	88.6 (73.0-98.9)	64.1 (47.2-79.0)
rC9	78.6 (59.0-91.7)	84.0 (65.28-94.4)
rA2	92.3 (74.9-99.0)	88.0 (73.8-95.9)

*a*: Se - sensitivity; *b*: 95% CI - 95% probability confidence interval; *c*: Sp - specificity

## DISCUSSION

Among the neglected tropical diseases, VL is one of the leading causes of mortality in Brazil and several other countries, especially among children and the elderly.^17^ Proper case detection for the reduction of mortality rates, identification of parasite hosts, and effective vector control strategies in endemic areas are mainstays of VL elimination programs. As such, rapid, sensitive, and inexpensive diagnostic tools capable of detecting VL in symptomatic and asymptomatic canine and human cases are essential.^18^

Although the development of tests based on the kinesin antigen represented significant progress in VL serodiagnosis, these tests failed to detect cases with low or absent levels of anti-*Leishmania*-specific antibodies, mainly in asymptomatic dogs, even if they were combined in a chimeric molecule (rK28).^7^ ^,^ ^19^ ^,^ ^20^ ^,^ ^21^ ^,^ ^22^ Therefore, there is a continuous search for new *Leishmania* antigens to develop more accurate diagnostic tests. Application of genomic and proteomic analyses have led to the identification of several other potential molecules for diagnosis of leishmaniasis, however, these candidate antigens require further validation.^13^

In this study, we tested and identified a new antigen, rFc (LinJ36_V3.0640), by comparing the previously described *Leishmania* spp. genomes^12^ as a proof of concept for the serodiagnosis of CVL and HVL. We further validated the potential of two other molecules, rC9 and rA2, which were previously tested against CVL and HVL sera.^10^ ^,^ ^14^ All three proteins were previously submitted to *in silico* prediction for application in immunoassays and displayed promising B cell epitopes.^13^ ^,^ ^15^

In ELISA for serodiagnosis of CVL, the sensitivities of rFc, rC9 and rA2 corresponded to 93.6%, 95.7%, and 93.6% with specificity values of 82.3%, 87.5%, and 81.2%, respectively. Similar sensitivity values were observed for the detection of antibodies anti-rFc, anti-rC9, and anti-rA2 in asymptomatic dogs (95%, 90%, 90%, respectively). High sensitivity values in this specific sample subset may be related to the small number of asymptomatic cases (n = 27) and to positive serological and parasitological results in all these animals. On the other hand, serological tests are generally less effective at detecting infection in asymptomatic animals, which may have low parasitism and produce low specific antibody titres.^23^

It is widely known that CVL diagnosis is a significant challenge for veterinarians, mainly in asymptomatic animals, given the limitations of serological and other non-invasive diagnostic tests and the lack of pathognomonic CVL signs.^22^ ^,^ ^23^ The low positive predictive and negative values of serological diagnostic tests impair the true positive diagnosis of dogs due to low sensitivity and cross-reactivity with other pathogens, contributing to the uncertainty of serological results.^24^ On the other hand, parasitological tests and PCR may lack, for several reasons, sensitivity in asymptomatic animals.^23^ ^)^ Consequently, we based our selection of positive animals on at least one positive result in direct microscopy or parasite culture and/or PCR and concordance with a positive serological result.

Although unavailable as a commercial test, the rA2 antigen has been shown to react with sera of dogs with VL from different geographic regions and, which is of special value, the diagnosis of asymptomatic cases.^10^ ^,^ ^11^ ^,^ ^20^ Moreover, a direct comparison between A2 and kinesin antigens (rK39 and rK26) utilising ELISA revealed a better performance of A2 among the asymptomatic animals.^11^ Farahmand et al.^20^ reported similar sensitivity values in symptomatic (52.9%) and asymptomatic dogs (53.5%) from Iran using an A2-ELISA. In addition, the A2-ELISA displayed the best sensitivity value for asymptomatic animals (53.5%) when compared to direct agglutination tests and the rKE16 dipstick. Akhoundi et al.,^25^ however, produced contradicting results when developing a latex agglutination test with rA2 (LAT-A2) and comparing it to a direct agglutination test with total promastigote antigens (PRO-DAT) to detect CVL cases from Iran; these results had a high degree of concordance between PRO-DAT and LAT-A2 as well as a high sensitivity of 95.2% for LAT-A2. As expected, the authors also noted that LAT-A2 allowed for the faster assessment of results in comparison to PRO-DAT, which is interesting with respect to field-testing and management of control measures in endemic areas.

**TABLE V:** Statistical analysis of enzyme-linked immunosorbent assay (ELISA) versus immunochromatographic tests (ICT) with rFc, rC9 and rA2, using canine and human sera

CVL - ELISA versus ICT		
Protein	Kappa index	value *p* ^*^
rFc	0.93	0.05
rC9	0.8	0.015
rA2	0.76	<0.001
HVL - ELISA versus ICT		
Protein	Kappa index	value *p* ^*^
rFc	0.69	0.08
rC9	0.65	< 0.001
rA2	0.98	1

CVL: canine visceral leishmaniasis; HVL: human visceral leishmaniasis: *: the McNemar test was used to estimate statistical differences between pairs of test. Differences were considered statistically significant when value p < 0.05.

**TABLE VI:** Statistical analysis of ICT-rFc, ICT-rC9 and ICT rA2 versus IFAT, IT-LEISH®, ELISAEXT, and ELISA-rK39, using human sera

Protein	ICT x IFAT	ICT x IT-LEISH^®^	ICT x ELISA_EXT_	ICT x ELISA-rK39
(kappa index / value *p* ^*^ )				
rFc	0.57 / < 0.001	0.47 / 0.002	0.53 / 0.014	0.50 / 0.006
rC9	0.60 / 0.45	0.54 / 0.75	0.60 / 0.54	0.58 / 0.77
rA2	0.74 / 0.579	0.67 / 0.75	0.74 / 1	0.77 / 0.449

*: McNemar test.

Of the three proteins, ELISA-rA2 displayed the best performance for identification of HVL, resulting in positive anti-rA2 antibody titres in 91.5% of the human positive-samples with a corresponded specificity of 87%. Since the first description of A2 as an HVL diagnostic antigen, studies have reported sensitivities varying from 60 to 92%, depending on the geographical origin of patients and the test format.^9^ ^,^ ^10^ In contrast, the rFc and rC9 proteins displayed better performance for the diagnosis of CVL over HVL, which, in the case of rC9, corroborated the data reported by Fonseca et al.^14^

ELISA is a widely used classical test that demonstrates the potential for the serological diagnosis of infectious diseases. Therefore, we initially chose ELISA for validating our candidate antigens before moving towards testing them with ICT. Transitioning from one test format to another is not simple and requires the optimisation of various key parameters for improved performance. ICT parameters included assay design, best conditions for antigen, reagents, membrane selection, equipment for the application of reagents onto membranes and precision cutting membranes, conjugation protocol, and others.

After adjustment of these parameters, anti-rFc, anti-rC9 and anti-rA2 antibodies were detected by ICTs in sera of *L. infantum*-infected dogs. The ICT-rFc, ICT-rC9, and ICT-rA2 displayed sensitivities/specificities of 88.6/84.0%, 86.5/92.0%, and 87/100%, respectively. The sensitivities of the ICTs were comparable to values previously reported with DPP^®^-CVL (86%) and sequential testing using DPP^®^-CVL and EIE^®^-CVL (73%).^26^ The Alere^TM^ test, another commercial ICT using the chimeric rK28 antigen, displayed sensitivities of 97.4% and 85.4%, depending on the previous screening test for defining the CVL sera panel.^21^ Thus, the sensitivity values obtained for the ICTs herein were consistent with results reported for other commercial tests; however, our tests were not developed in an industrial setting, and further improvements may still be implemented.

The ICT-rA2 displayed very good performance (sensitivity of 92.3% and specificity of 88%) and high concordances for HVL diagnosis with IFAT (k = 0.74), IT-LEISH^®^ (k = 0.67), ELISA_EXT_ (k = 0.74) and ELISA-rK39 (k = 0.77). No statistical difference was observed with ELISA-rA2 when applied to the same set of samples. Therefore, the ICT-rA2 was compatible both with the “in house” ELISA and the commercial immunochromatographic tests (IT-LEISH^®^) described above. In contrast, the ICT-rFc and ICT-rC9 displayed inconsistency with values of 88.6% and 78.6% for sensitivity and 64.1% and 84% for specificity, respectively.

Interestingly, 92% of VL patient sera from Rio Grande do Norte also displayed cross-reactivity with a commercial ELISA test for Chagas disease. This result illustrates the difficulties clinicians face in distinguishing infections and/or parasite exposure by *Leishmania* and *Trypanosoma cruzi* in patients living in overlapping transmission areas. Indeed, transmission areas of *T. cruzi* and *Leishmania* species other than *L. infantum* also significantly overlap in Brazil. Thus, false positive results with the commercial ELISA Chagas disease test may result from exposure to antigens of *L. infantum* or other species that cause ATL.^27^ ^)^ Cross-reactivity may also be expected due to significant homology among *Leishmania* spp. and *T. cruzi* protein sequences.^27^ Protein blast searches of the rC9, rFc, and rA2 amino acid sequences against the *T. cruzi* protein database revealed 50% identity between C9 and a hypothetical protein, 46% identity between rFc and its *T. cruzi* ortholog protein, and 31% identity between A2 and trans-sialidase. These results suggest potential, but low, cross-reactivity with *T. cruzi* antigens. This finding may explain the reactivity of rFc with four out of five sera samples of patients with Chagas disease. Nonetheless, specific recombinant protein-based serodiagnosis may overcome these drawbacks by eliminating cross-reactivity induced by the highly conserved antigens shared by *Leishmania* spp. and *T. cruzi*.

In the context of epidemiological surveys, immunochromatographic tests used for screening seropositive individuals should ideally display high sensitivity, whereas highly sensitive and specific tests must confirm positive results.^28^ Considering the heterogeneity of the MHC molecules of healthy individuals, chimeric antigens are expected to amplify epitope recognition and provide more sensitive tests. Thus, the antigens tested herein may be added to chimeric proteins for improved performance. However, given the already high concordance, as shown in the Venn diagrams presented here (Fig. 5), there is no strong indication that the combination of the rA2, rFc, and rC9 antigens would significantly improve the sensitivity of ELISA for CVL diagnosis. On the other hand, for human ELISA, the combination of rA2 and rC9 may improve sensitivity. In an IC test, a combination of rA2 and rFc would improve sensitivity either for CVL or HVL.

Another important issue regarding serological diagnosis of dogs is the cross-reactive antibodies that are introduced by vaccination. The Leish-Tec^®^ vaccine, licensed in Brazil for use in dogs as an individual protective measure, is composed of saponin and the rA2 protein. Leish-Tec^®^ is a DIVA (Differentiates Infected and Vaccinated Animals) vaccine, allowing for the differentiation of the antibody responses due to infection from those due to the vaccination.^29^ ^,^ ^30^ Although the rA2 antigen is a promising candidate antigen for screening or confirmatory tests, it has also been associated with the antibody responses induced by the Leish-Tec^®^ vaccine.^30^ Therefore, a diagnostic test for the detection of CVL in Leish-Tec^®^-vaccinated dogs may require another antigen to detect antibodies induced by infection.

In conclusion, the present study demonstrated that the rA2, rC9, and rFc antigens are promising antigens for VL diagnosis, regardless of the test format. It is worth mentioning that both ELISA and ICT assays were developed in a research laboratory setting and tested on a small scale. Other limitations of this study are the predominant origin of dogs from the Centres of Zoonosis Control and the lack of results of parasitological tests for part of the samples that constituted the human sera panel. Although positive results in more than one serological test were used as criteria for HVL diagnosis and to set the HVL sera panel, serological tests may display low positive and negative predictive values, impacting the evaluation of sensitivity and specificity of the diagnostic assays. Further improvements in the serological assays may require their combination for increased sensitivity, production under optimised conditions according to industrial requirements, or testing with larger and better-characterised panels of canine and human sera samples.
